# Normative values and predictive equations of hand-held dynamometry for upper limb muscles in healthy adults

**DOI:** 10.1590/1414-431X2026e15416

**Published:** 2026-08-21

**Authors:** B.R.V. Nepomuceno, F.T.O De Oliveira, I.F.S.R. Schindler, M.B. Machado, M. Gomes-Neto

**Affiliations:** 1 Programa de Pós-Graduação em Processos Interativos de =rgãos e Sistemas, Universidade Federal da Bahia, Salvador, BA, Brasil; 2 Escola Bahiana de Medicina e Saúde Pública, Salvador, BA, Brasil; 3 Grupo de Pesquisa em Fisioterapia, Universidade Federal da Bahia, Salvador, BA, Brasil

**Keywords:** Hand-held dynamometry, Muscle strength, Reference values, Predictive equations, Upper extremity

## Abstract

The aim of this study was to establish comprehensive normative values and generate robust predictive equations for isometric upper limb muscle strength using hand-held dynamometry (HHD) in healthy adults aged 18 to 80 years. A cross-sectional multicenter study was conducted with 210 healthy volunteers aged 18 to 80 years. Participants were rigorously screened to exclude cognitive impairment, acute musculoskeletal symptoms, or systemic conditions that could influence strength. Participants underwent standardized assessment of isometric strength using a handheld dynamometer for 10 key upper limb muscle groups (dominant and non-dominant limbs), and maximal isometric strength (kgf) of each muscle group was recorded. Primary analytical outcomes included age- and sex-specific reference values and derived predictive equations for strength. Novel predictive equations were successfully developed for all 10 upper limb muscle groups from this diverse sample. A very good correlation (r^2^ range: 0.62-0.77, P<0.001) was observed between direct HHD measurements and the proposed equations. Importantly, these new equations demonstrated superior predictability for this population compared to previously published North American reference equations. This study provides the first comprehensive set of age- and sex-specific normative values and predictive equations for isometric upper limb muscle strength via HHD in healthy adults. These invaluable normative data enable rehabilitation professionals to objectively assess strength, identify weakness early, and enhance treatment monitoring, thus strengthening evidence-based practice in physical medicine and rehabilitation.

## Introduction

For a long time, the musculoskeletal system has been viewed solely as a system responsible for locomotion. In recent years, evidence has shown that the overall health of this tissue is associated with the body's homeostasis, as well as immunological balance and energy reserves (1-4). Recent studies have shown that good muscle tropism is related to lower morbidity and mortality in individuals who are hospitalized (5-8), highlighting the importance of the musculoskeletal system.

However, despite the recognition of the importance of muscle health and the preservation of this tissue during illness, methods for evaluating strength - the main component of muscle homeostasis - remain limited to those that categorize rather than quantify strength (9). The psychometric properties of hand-held dynamometry (HHD) for the upper limbs have been systematically studied in recent years (10,11).

Defining the normal ranges of muscle strength in the major muscles of the upper limbs is necessary for the early identification of individuals with muscle weakness and for implementing interventions to reverse this condition.

This study aimed to establish normative values and a predictive equation for isometric strength of upper limb muscles in healthy individuals aged 18 to 80 years.

## Material and Methods

A cross-sectional study evaluated muscle strength measured by HHD in healthy individuals aged between 18 and 80 years, of both sexes, who were clinically free of neuromuscular diseases.

### Sample size

A model was developed to determine the sample size within a 95% confidence interval of the mean, with a margin of error not exceeding 10% of the peak torque values and an estimated sample loss of 10%. Accordingly, 30 individuals per age subgroup were stipulated, resulting in a total sample of 210 participants.

### Sample selection

The study sample was composed of self-reported healthy Brazilian volunteers aged between 18 and 80 years who signed the informed consent form. Individuals were excluded if they were cognitively incapable of understanding the protocol and instructions; had osteoarticular symptoms within the last two months; presented neuromuscular dysfunctions, even if not acute, associated with a reduced functional range of motion; had any cardiorespiratory or systemic condition that could contraindicate the muscle strength evaluation protocol; or had used stimulant substances that could interfere with isometric strength, such as anabolic steroids, in the last 6 months (11-13).

### Measurement log

The evaluation of isometric muscle strength was performed using a handheld dynamometer (HHD) (model 01165, Lafayette Instrument Co., USA). A goniometer (Instituto São Paulo, Brazil) was used to ensure adequate marking of the joint position for testing each muscle group. The evaluation was conducted in a reserved and appropriate setting. Isometric contraction was tested for the main movements of the major joints of the upper limbs, considering both the dominant (d) and non-dominant (n-d) limbs. Volunteers were oriented, before the measurement, regarding the study protocol and possible discomforts occurring during and after the exercise. Training and warm-up were performed for each movement before testing.

Isometric contraction was performed for each movement for 3 s and was stopped when the equipment beeped. Each movement was performed in three repetitions, with the highest value considered, and always under the encouragement of the examiner: “Push, push, push!” (14,15).

**Figure 1:**
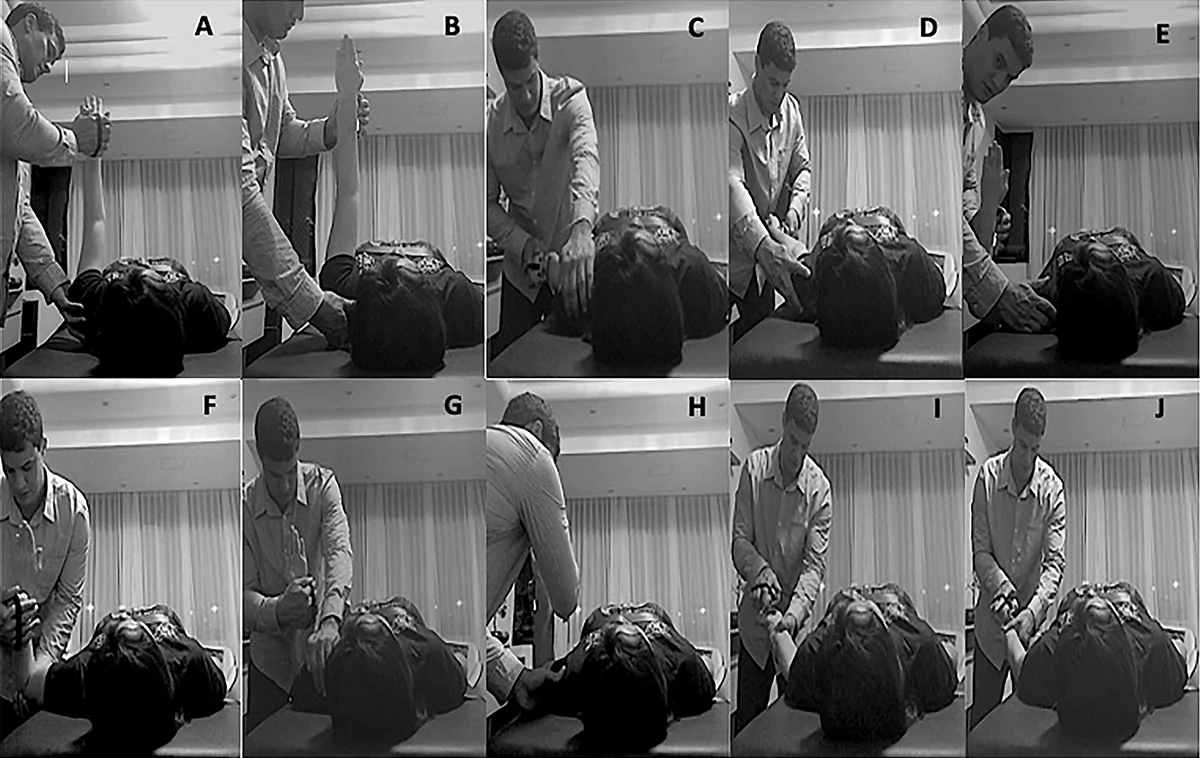
Positioning of the participants for strength assessment.

A total of 10 muscle groups were evaluated for the main functional movements of the upper limbs in the following positions: 1) Shoulder flexors: in dorsal decubitus, with the shoulder positioned at 90° of flexion, elbow and wrist at 180°, and forearm pronated; the dynamometer was supported on the anterior surface of the arm, with an indication of shoulder flexion. 2) Shoulder extensors: in dorsal decubitus, with the shoulder positioned at 90° of flexion, elbow and wrist at 180°, and forearm pronated; the dynamometer was supported on the anterior surface of the arm, with an indication of shoulder extension. 3) Shoulder abductors: in dorsal decubitus, with the shoulder positioned at 45° of abduction, elbow and wrist at 180°, and forearm pronated; the dynamometer was supported on the lateral surface of the arm, with an indication of shoulder abduction. 4) Shoulder adductors: in dorsal decubitus, with the shoulder positioned at 45° of abduction, elbow and wrist at 180°, and forearm pronated; the HHD was supported on the medial surface of the arm, with an indication of shoulder adduction. 5) Internal rotators: in dorsal decubitus, with the shoulder at 90° of abduction, elbow at 90° of flexion, wrist at 180°, and forearm pronated; the dynamometer was supported on the anterior surface of the wrist, with an indication of internal rotation. 6) Shoulder external rotators: in dorsal decubitus, with the shoulder at 90° of abduction, elbow at 90° of flexion, wrist at 180°, and forearm pronated; the dynamometer was supported on the posterior surface of the wrist, with an indication of external rotation. 7) Elbow flexors: in dorsal decubitus, with the shoulder in a neutral position, elbow at 90° of flexion, wrist at 180°, and forearm supinated; the dynamometer was positioned to indicate elbow flexion. 8) Elbow extensors: in dorsal decubitus, with the shoulder in a neutral position, elbow at 90° of flexion, wrist at 180°, and forearm supinated; the HHD was supported on the posterior surface of the wrist, with an indication of elbow extension. 9) Wrist flexors: in dorsal decubitus, with the forearm supported on a rigid surface and the wrist beyond the edge of the surface; the shoulder was positioned at 0° of flexion, elbow at 90° of flexion, wrist at 180°, and forearm supinated; the dynamometer was supported on the anterior surface of the carpal bones, with an indication of wrist flexion. 10) Wrist extensors: in dorsal decubitus, with the forearm supported on a rigid surface and the wrist beyond the edge of the surface; the shoulder was positioned at 0° of flexion, elbow at 90° of flexion, wrist at 180°, and forearm pronated; the HHD was supported on the posterior surface of the carpal bones, with an indication of wrist extension. The measurement positions are illustrated in Figure 1.

To ensure maximum torque for each muscle group, the HHD was positioned by the examiner’s hands on the evaluated segment, always applying sufficient resistance to prevent the requested movement. All measuring instruments were calibrated prior to the study to minimize measurement bias.

### Ethical precepts

The research was approved by the Research Ethics Committee of the Institute of Health Sciences of the Federal University Of Bahia prior to the study (No. 1.537.948). The integrity and confidentiality of the volunteers’ individual data were guaranteed in accordance with the Declaration of Helsinki.

### Statistical analysis

The Statistical Package for the Social Sciences software for Mac (version 28.0, IBM, USA) was used for data tabulation and analysis. Descriptive statistics were used, with data presented in tables and graphs. Qualitative data are reported as absolute and relative frequencies, while quantitative data are reported as mean and standard deviation and/or minimum and maximum.

### Linear regression and validation

The definition of normative values for muscle strength using the HHD was performed within the 95% confidence interval based on Student's *t*-test, with dichotomization by sex. The linear regression model was used to identify variables with a coefficient of determination (r^2^) for muscle strength and statistical significance for inclusion in the equation for each muscle group, to calculate the reference equation for muscle strength measured by HHD for each group, considering P<0.05 as significant. Furthermore, analysis of variance (ANOVA) was used to determine constants and r^2^ between each variable and the strength of the muscle group in question. The equation was then applied to the test population and correlated with the actual HHD values using Pearson's correlation coefficient (r) to validate the equation. The correlation was categorized according to the classification proposed by Weir: excellent (1.0 to 0.81), very good (0.80 to 0.61), good (0.60 to 0.41), reasonable (0.40 to 0.21), and poor (0.20 to 0.00). The HHD correlation for elbow flexion and shoulder extension was compared with the previously proposed North American predictor equation and the predictor equation proposed in this study for comparative purposes.

## Results

**Figure 2:**
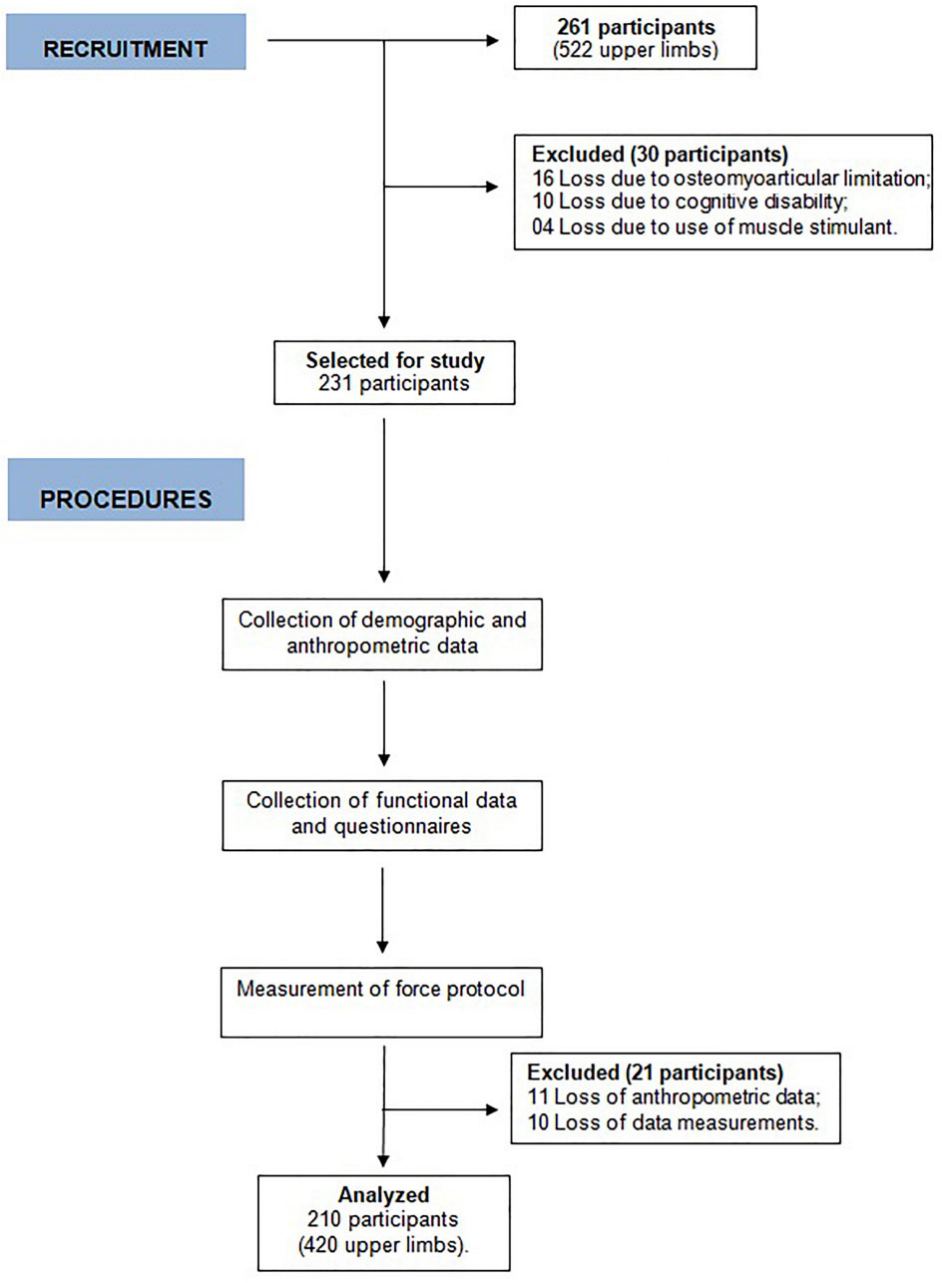
Flowchart of patient enrollment in the study.

A total of 210 volunteers (Figure 2), representing 11 Brazilian states, most from the Northeast region, participated in the study. Participants ranged in age from 18 to 80 years, with a mean height of 1.68 m and a mean weight of 71.6 kg. The most frequent race was Black (41.9% of volunteers), and 54% of the individuals had received higher education. Regarding physical activity, as categorized by the International Physical Activity Questionnaire (IPAQ), 45% of the volunteers were irregularly active, and almost 22% were sedentary. Only 18.6% followed a diet; balanced and hyperprotein diets were the most common among study participants (Table 1).

**Table 1:** Sociodemographic, physical, and functional variables of the sample (n=210).

	f (%)	Mean (SD)
Sex, women	112 (53.3%)	
Age, years		46.2 (19.1)
Height, meters		1.68 (0.1)
Weight, kg		71.6 (14.7)
Race		
Black	88 (41.9)	
Brown	56 (26.7)	
White	50 (23.8)	
Others	16 (7.6)	
Region of the country		
Northeast	102 (48.6)	
North	47 (22.4)	
Southeast	25 (11.9)	
South	23 (10.9)	
Midwest	13 (6.2)	
Sleep, hours		6.6 (1.2)
IPAQ		
Irregularly active	96 (45.7)	
Sedentary	46 (21.9)	
Active	43 (20.5)	
Very active	25 (11.9)	
Frequency of physical activity (per week)		3.5 (2.0)
Type of physical activity		
Bodybuilding	57 (39.9)	
Cross-training	28 (19.6)	
Martial arts	15 (10.5)	
Running	12 (8.4)	
Pilates	10 (7.0)	
Others	21 (14.6)	
Regimen, yes	39 (18.6)	

IPAQ: International Physical Activity Questionnaire.

Supplementary Table S1 presents normative strength values by age group and sex for shoulder movements. Supplementary Table S2 presents these values for the elbow and wrist. Supplementary Table S3 shows the development of reference equations for maximum isometric strength for 10 movements of the dominant upper limb, and Supplementary Table S4 provides these equations for the non-dominant upper limb.

The following reference equations were created for the isometric strength of the muscle groups studied based on the sample evaluated: Shoulder flexors HHD d = 69.51 − (0.45×Age)+(0.38×Weight)+(33.35, if male) or HHD n-d = 47.68 − (0.58×Age)+(0.77 x Weight)+(35.62 if male); Shoulder extensors HHD d = 80.58 − (0.56×Age)+(0.39×Weight)+(45.61, if male), or HHD n-d = 81.04 − (0.47×Age)+(0.29×Weight)+(46.90, if male); Shoulder abductors HHD d = 50.40 − (0.51×Age)+(0.72×Weight)+(33.27, if male) or HHD n-d = 46.10 − (0.43×Age)+(0.67×Weight)+(36.01, if male); Shoulder adductors HHD d = 69.59 − (0.51×Age)+(0.40×Weight)+(39.69, if male) or HHD n-d = 75.98 − (0.50×Age)+(0.27×Weight)+(35.64, if male); Internal rotators of shoulder HHD d = 88.25 − (0.51×Age)+(0.33×Weight)+(39.67, if male) or HHD n-d = 83.92 − (0.46×Age)+(0.39×Weight)+(41.94, if male); External shoulder rotators HHD d = 77.73 − (0.37×Age)+(0.30×Weight)+(33.78, if male) or HHD n-d = 77.90 − (0.39×Age)+(0.35×Weight)+(30.99, if male); Elbow flexors HHD d = 151.95 − (0.78×Age)+(0.28×Weight)+(54.15, if male) or HHD n-d = 144.03 − (0.79×Age)+(0.34×Weight)+(53.61, if male); Elbow extensors HHD d = 82.31 − (0.50×Age)+(0.43×Weight)+(36.67, if male) or HHD n-d = 78.88 − (0.50×Age)+(0.44×Weight)+(35.70, if male); Wrist flexors HHD d = 65.11 − (0.40×Age)+(0.38×Weight)+(25.40, if male) or HHD n-d = 65.41 − (0.21×Age)+(0.34×Weight)+(24.40, if male); Wrist extensors HHD d = 63.95 − (0.47×Age)+(0.48×Weight)+(20.40, if male) or HHD n-d = 64.03 − (0.37×Age)+(0.42×Weight)+(17.17, if male).

**Figure 3:**
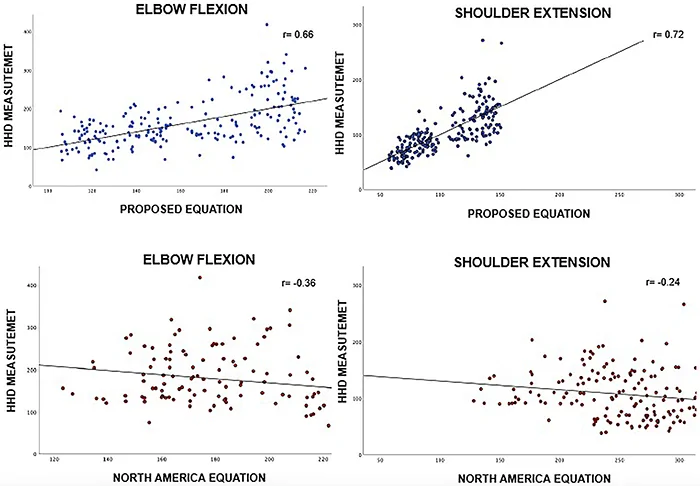
Correlation between muscle strength measured by hand-held dynamometry (HHD) and reference values for the North American population.

The comparison between the direct measurement of HHD and the proposed strength equation revealed a very good correlation. The correlation magnitudes were as follows: shoulder flexion (r^2^=0.76, P<0.001); shoulder extension (r^2^=0.74, P<0.001); shoulder abduction (r^2^=0.72, P<0.001); shoulder adduction (r^2^=0.62, P<0.001); shoulder internal rotation (r^2^=0.65, P<0.001); shoulder external rotation (r^2^=0.63, P<0.001); elbow flexion (r^2^=0.66, P<0.001); elbow extension (r^2^=0.69, P<0.001); wrist flexion (r^2^=0.77, P<0.001); and wrist extension (r^2^=0.72, P<0.001). Figure 3 shows the correlation between isometric strength measured with HHD for the sample, the predictor equation proposed in this study, and the North American equation proposed in a previous study (1).

## Discussion

This study established normative values for isometric muscle strength and developed reference equations to predict this variable in self-reported healthy individuals aged 18 to 80 years for 10 major upper limb muscle groups. No previous studies in the literature have defined normal values for isometric strength in upper limb muscle groups among adults of both sexes, aged 18 to 80 years, in the South American population. This highlights the importance of this study in identifying the magnitude of muscle strength in healthy adults and enables the classification of upper limb isometric strength as normal or weak in healthy adults aged 18 to 80 years.

This research represents a pioneering effort in the detailed characterization of major upper limb muscle groups. Muscle strength is increasingly recognized in clinical settings as a robust prognostic indicator for key outcomes, including mortality, morbidity, and hospitalization risk. The established reference values enable a more thorough evaluation of musculoskeletal health stratified by age (1,3,4).

Bohannon (16), in a previous study using a methodology similar to that used for North American adults aged 20 to 79 years (mean of 21 individuals per subgroup), described reference values and predictive equations for isometric strength for 10 muscle groups, 6 of which were in the upper limbs (abduction, external rotation, shoulder extension, elbow flexion and extension, and wrist extension). In this study, a correlation was made between the North American equations and the HHD results for this population, which were lower than those found using the equation proposed in this study.

In 1911, van der Ploeg et al. (17) described isometric force with HHD (with a maximum capacity of 250 N) in 100 Dutch individuals aged 20 to 60 years. They measured 13 muscle groups in the trunk and limbs, focusing on four upper limb movements: shoulder abduction (from 72 to 142 N for women and from 97 to 232 N for men), elbow flexion (from 127 to >250 N for women and from 193 to >250 N for men), elbow extension (from 68 to 141 N for women and from 88 to 225 N for men), and wrist extension (from 77 to 160 N for women and from 118 to 214 N for men). In that study, strength was categorized only by sex, not by age group, which limited the extrapolation of the data to other populations.

Andrews et al. (18) evaluated the isometric strength of 147 North American volunteers, 77 males and 70 females, aged 50 to 79 years. Thirteen muscle groups were measured, including flexion, extension, abduction, internal and external rotation of the shoulders, flexion and extension of the elbow, and extension of the wrist. In that study, no fixations were used on proximal segments to stabilize and prevent compensations, and the results for both sexes in equivalent age categories were much higher than the average strength found in the current study.

McKay et al. (19), in a study with 1,000 Australian individuals aged 3 to 101 years, described reference values for isometric strength using HHD. Participants were categorized by life stage: 3-9, 10-19, 20-59, and >60 years. The internal and external rotators of the shoulder, as well as the flexors and extensors of the elbow, were evaluated in the upper limbs among the 12 muscle groups analyzed.

Conversely, Hébert et al. (20) measured mean isometric strength in American children and adolescents aged 4 to 17 years across 10 movements, of which only 4 involved the upper limbs: shoulder abduction (mean 0.33 Nm/kg at 4 years to 0.89 Nm/kg at 17 years for females and 0.34 Nm/kg at 4 years to 1.13 Nm/kg at 17 years for males), external rotation of the shoulder (mean 0.26 Nm/kg at 4 years to 0.34 Nm/kg at 17 years for females and 0.30 Nm/kg at 4 years to 0.51 Nm/kg at 17 years for males), elbow flexion (mean 0.33 Nm/kg at 4 years to 0.68 Nm/kg at 17 years for females and 0.33 Nm/kg at 4 years to 1.08 Nm/kg at 17 years for males), and elbow extension (mean 0.38 Nm/kg at 4 years to 0.46 Nm/kg at 17 years for females and 0.40 Nm/kg at 4 years to 0.68 Nm/kg at 17 years for males). The authors included 328 individuals, with 13 individuals per group. In this current study, the age range was adults between 18 and 80 years, with 30 individuals per group. Hébert et al. (20) did not create a reference equation for the strength values found in their sample, as was done in this study.

The current study used a protocol that is easily reproducible, based on previous research on measurement reliability and validation of the proposed method. A key strength of this protocol is its versatility, allowing it to be applied in various settings with low cost and adequate reproducibility. In a previous study by our group (13), this protocol was used in a hospital environment with intensive care patients, confirming its safety.

The limitations of this study include the unbalanced number of individuals across the centers where the research was conducted, due to the recent pandemic, compromising the possibility of achieving balance between the centers. Another potential limitation is the study population, as individuals over 80 years were not included in the analysis, as well as the study's ability to track the percentage of lean body mass. Other variables that could influence muscle performance in the strength test include the lack of tracking of muscle anabolic substance use, such as caffeine or other supplements; the absence of detailed reporting of weekly physical activity in hours; and missing information on the volunteers' biotype, all of which could also be related to maximum isometric strength. This study did not test the predictor equations in an independent sample and encourages future research to test these equations in samples from various locations in Brazil, among practitioners of specific sports modalities, and among patients.

## Conclusion

This study established normative values for isometric strength of the main muscle groups of the upper limbs and developed equations to predict the strength of these groups in individuals aged 18 to 80 years. The results are expected to guide future publications on muscle strength levels in other populations and may enable earlier diagnosis and treatment of muscle weakness.

## Data Availability

The datasets generated and/or analyzed during the current study are available from the corresponding author on reasonable request.

## Supplementary Materials

Click to view [pdf].
