# Supplementary material for: Normative values and predictive equations of hand-held dynamometry for upper limb muscles in healthy adults
Source: Braz J Med Biol Res. 2026 Aug 21;59:e15416. doi: 10.1590/1414-431X2026e15416 (PMC13502766; doi:10.1590/1414-431X2026e15416)
Supplement: Supplementary file 1 [file 1414-431X-bjmbr-59-e15416-suppl.pdf]

Table S1. Reference values in Newtons for muscle strength measured using hand-held dynamometry for shoulder muscles (n=210).

		Shoulder – Action											
Age group		Flexion		Extension		Abduction		Adduction		Internal rotation		External rotation	
		Men (n=15)	Women (n=15)	Men (n=15)	Women (n=15)	Men (n=15)	Women (n=15)	Men (n=15)	Women (n=15)	Men (n=15)	Women (n=15)	Men (n=15)	Women (n=15)
18–20 (n=30)	95%CI	92.10–128.99	70.24–87.24	113.86–163.30	78.58–102.59	96.87–130.93	71.90–94.75	97.01–147.68	64.16–87.02	116.57–156.83	78.01–102.43	110.65–154.99	74.68–102.10
	$\bar{x}$(se)	110.54 (8.60)	78.74 (3.96)	138.58 (11.52)	90.58 (5.59)	113.89 (7.93)	83.32 (5.32)	122.34 (11.81)	75.58 (5.32)	136.70 (9.38)	90.22 (5.69)	132.82 (10.33)	88.39 (6.39)
21–30 (n=30)	95%CI	108.65–131.57	74.44–90.31	117.92–140.83	79.78–100.22	110.86–140.02	71.63–85.17	100.02–138.19	65.50–88.57	115.83–137.63	78.07–98.50	102.93–125.89	73.19–87.63
	$\bar{x}$(se)	120.11 (5.43)	82.37 (3.60)	129.37 (5.42)	90.00 (4.64)	125.43 (6.91)	78.40 (3.07)	119.10 (9.04)	77.03 (5.23)	126.72 (5.16)	88.28 (4.64)	114.41 (5.43)	80.40 (3.27)
31–40 (n=30)	95%CI	128.78–144.60	80.90–95.76	145.86–185.15	83.52–108.18	129.40–170.70	73.45–102.29	124.39–175.28	76.02–99.97	137.95–198.66	89.64–108.93	123.39–157.77	81.20–98.87
	$\bar{x}$(se)	136.68 (3.72)	88.33 (3.41)	165.50 (9.26)	95.84 (5.65)	150.05 (9.74)	87.86 (6.61)	149.83 (12.00)	87.99 (5.49)	168.30 (14.31)	99.28 (4.42)	140.58 (8.10)	90.03 (4.05)
41–50 (n=30)	95%CI	92.00–123.12	65.84–80.62	108.63–144.65	73.67–89.46	94.55–127.91	65.99–82.70	94.86–153.97	64.35–83.30	106.40–164.18	77.03–102.38	82.67–144.22	72.81–84.04
	$\bar{x}$(se)	107.56 (7.14)	74.72 (4.19)	126.63 (8.26)	81.56 (3.72)	111.23 (7.65)	74.34 (3.94)	124.41 (13.56)	73.82 (4.46)	135.29 (13.25)	89.70 (5.97)	113.44 (14.12)	78.42 (2.64)
51–60 (n=30)	95%CI	97.35–116.46	67.31–80.63	102.52–131.72	67.19–85.90	95.32–134.15	63.37–75.24	92.50–108.28	57.33–74.66	101.66–132.31	78.68–97.96	91.22–112.58	70.29–86.74
	$\bar{x}$(se)	106.90 (4.45)	73.96 (3.10)	117.12 (6.80)	76.54 (4.36)	114.73 (9.05)	69.30 (2.76)	100.38 (3.67)	65.99 (4.04)	116.98 (7.14)	88.32 (4.49)	101.90 (4.97)	78.51 (3.83)
61–70 (n=30)	95%CI	81.20–119.18	57.21–69.00	109.60–161.68	60.23–74.74	90.05–111.53	57.76–69.77	77.67–128.41	60.95–73.74	103.00–147.70	64.79–80.03	83.79–121.61	68.66–83.87
	$\bar{x}$(se)	100.19 (8.39)	63.10 (2.81)	135.64 (11.51)	67.48 (3.46)	100.79 (4.74)	63.76 (2.86)	103.04 (11.21)	67.34 (3.05)	125.35 (9.87)	72.41 (3.63)	102.70 (8.36)	76.26 (3.63)
71–80 (n=30)	95%CI	77.41–112.73	52.43–65.61	89.41–116.46	56.40–70.40	76.00–118.22	53.84–72.30	76.19–107.39	49.50–64.65	76.24–109.70	65.60–85.53	78.25–125.99	75.92–82.64
	$\bar{x}$(se)	95.07 (7.80)	59.02 (3.14)	101.43 (6.64)	63.40 (3.34)	87.11 (13.75)	63.07 (4.40)	91.79 (6.89)	57.08 (3.61)	93.05 (7.43)	75.56 (4.76)	116.95 (12.68)	68.79 (4.27)

Table S2. Reference values for muscle strength measured using hand-held dynamometry for elbow and wrist muscles (n=210).

		Elbow – Action				Wrist – Action			
		Flexion		Extension		Flexion		Extension	
Age group		Men (n=15)	Women (n=15)	Men (n=15)	Women (n=15)	Men (n=15)	Women (n=15)	Men (n=15)	Women (n=15)
18–20 (n=30)	95%CI	189.08–244.76	122.81–170.35	122.71–162.36	79.11–111.66	102.56–135.21	62.56–85.47	98.05–129.83	65.37–100.12
	$\bar{x}$(se)	216.92 (12.98)	146.58 (11.08)	142.54 (9.24)	95.38 (7.58)	118.88 (7.61)	74.02 (5.33)	113.94 (7.40)	82.75 (8.10)
21–30 (n=30)	95%CI	154.66–211.99	127.26–151.52	102.62–134.81	77.04–110.67	85.57–114.83	66.83–95.59	84.05–105.83	63.98–87.26
	$\bar{x}$(se)	183.32 (13.58)	139.39 (5.51)	118.71 (7.62)	93.85 (7.63)	100.20 (6.93)	81.21 (6.53)	94.94 (5.16)	75.62 (5.28)
31–40 (n=30)	95%CI	200.01–280.95	127.87–170.36	133.79–201.81	86.35–106.22	88.09–145.96	69.74–100.37	96.15–162.44	70.40–100.58
	$\bar{x}$(se)	240.48 (19.09)	149.12 (9.75)	167.80 (16.04)	96.29 (4.55)	117.02 (13.64)	85.06 (7.02)	129.30 (15.63)	85.49 (6.92)
41–50 (n=30)	95%CI	168.77–243.32	118.44–158.65	106.08–180.77	70.98–92.30	81.12–122.03	58.17–80.96	75.94–139.71	62.21–84.03
	$\bar{x}$(se)	206.05 (17.10)	138.55 (9.48)	143.43 (17.14)	81.64 (5.02)	101.57 (9.38)	69.57 (5.37)	107.83 (14.63)	73.12 (5.14)
51–60 (n=30)	95%CI	131.60–203.69	104.67–137.76	92.24–134.31	70.57–99.86	71.99–104.97	58.63–74.36	68.20–110.20	54.39 - 73.48
	$\bar{x}$(se)	167.64 (16.80)	121.22 (7.71)	113.28 (9.80)	85.22 (6.82)	88.48 (7.68)	66.50 (3.66)	89.20 (9.79)	63.4 (4.45)
61–70 (n=30)	95%CI	142.37–210.92	104.92–137.62	87.25–132.44	70.71–95.50	75.25–132.10	59.00–74.46	65.63–114.70	64.77–82.32
	$\bar{x}$(se)	176.65 (15.15)	121.27 (7.81)	109.85 (9.98)	83.11 (5.92)	103.68 (12.56)	66.73 (3.69)	90.17 (10.84)	73.55 (4.19)
71–80 (n=30)	95%CI	127.68–165.47	104.39–138.35	84.47–123.54	66.09–85.12	71.27–93.42	50.17–69.93	55.18–89.49	50.07–73.94
	$\bar{x}$(se)	146.58 (8.35)	121.37 (8.11)	104.01 (8.63)	75.61 (4.54)	82.35 (4.89)	60.05 (4.72)	72.34 (4.40)	62.01 (5.70)

Table S3. Development of the predictive equation model of maximum isometric strength for dominant upper limb muscles from multiple linear regression (n=210).

Models	r	r^2	$r^{2\ adjusted}$	SEE	P	ß	Age	Sex	Weight	Frequency of activity	Height	Activity	Sleep time
Shoulder–flexion													
Model 1	0.769	0.592	0.570	19.990	<0.001	80.090	–0.533*	32.415*	0.408*	0.550	0.015	6.966	–0.704
Model 2	0.776	0.598	0.571	19.656	<0.001	69.514	–0.445*	33.348*	0.378*	–	–	–	–
Extension													
Model 1	0.771	0.594	0.572	25.833	<0.001	112.586	–0.599*	42.574*	0.470*	0.811	–0.170	1.434	–2.511
Model 2	0.793	0.592	0.585	26.642	<0.001	80.580	–0.563*	45.611*	0.393*	–	–	–	–
Abduction													
Model 1	0.753	0.567	0.543	25.312	<0.001	71.707	–0.490*	34.114*	0.795*	2.048	0.055	5.794	–1.163
Model 2	0.773	0.572	0.565	25.307	<0.001	50.400	–0.507	33.267*	0.719*	–	–	–	–
Adduction													
Model 1	0.723	0.523	0.497	29.203	<0.001	109.274	–0574*	39.769*	0.543*	5.077†	–0.44	1.266	–1.059
Model 2	0.757	0.531	0523	30.345	<0.001	69.592	–0.507*	39.690*	0.396*	–	–	–	–
Internal rotation													
Model 1	0.718	0.515	0.484	31.054	<0.001	143.335	–0.487*	38.878*	0.582*	6.621†	–0.153	0.081	3.303
Model 2	0.734	0.522	0.513	31.372	<0.001	88.245	–0.506*	39.667*	0.326*	–	–	–	–
External rotation													
Model 1	0.677	0.459	0.430	26.218	<0.001	122.920	–0.374*	33.483*	0.402*	5.102†	–0.080	3.717	–2.847
Model 2	0.728	0.494	0.485	26.661	<0.001	77.729	–0.369*	33.782*	0.297*	–	–	–	–
Elbow–Flexion													
Model 1	0.700	0.490	0.463	42.764	<0.001	266.010	–0.761*	48.410*	0.476*	12.848†	–0.332	11.551	–5.485
Model 2	0.715	0.492	0.466	49.475	<0.001	151.949	–0.776*	54.149*	0.280*	–	–	–	–
Extension													
Model 1	0.647	0.418	0.387	35.927	<0.001	147.543	–0.460*	36.638*	0.560*	8.107†	–0.070	1.517	–5.208
Model 2	0.665	0.420	0.410	36.592	<0.001	82.311	–0.495*	36.670*	0.426*	–	–	–	–
Wrist–Flexion													
Model 1	0.638	0.407	0.375	28.138	<0.001	122.507	–0.454*	25.602*	0.469*	6.102†	–0.152	6.900	–3.032
Model 2	0.643	0.415	0.402	30.227	<0.001	65.107	–0.395*	25.398*	0.377*	–	–	–	–
Extension													
Model 1	0.665	0.442	0.412	29.334	<0.001	121.449	–0.470*	19.419*	0.667*	14.446	–0.001	14.446	–0.787
Model 2	0.682	0.447	0.441	32.901	<0.001	63.947	–0.456*	20.392*	0.476*	–	–	–	–

*P<0.05; †Variable with P>0.05 with the removal of regression; Model 1: Initial regression model containing variables with potential to determine the peak torque of isometric contraction in the literature; Model 2: Regression model keeping only variables with P<0.05 in model 1; r: Correlation coefficient; r^2: Coefficient of determination; $r^{2adjusted}$: Coefficient of determination adjusted for chance; SEE: Estimated standard error; ß: Standardized coefficient.

Table S4. Development of the predictive equation model of maximum isometric strength of non-dominant upper limb muscles from multiple linear regression (n=210).

Models	r	r^2	$r^{2\ adjusted}$	SEE	P	ß	Age	Sex	Weight	Frequency of activity	Height	Activity	Sleep time
Shoulder–flexion													
Model 1	0.599	0.459	0.425	23.545	<0.001	18.863	–0.695*	33.940*	0.892*	1.109	0.028	2.818	2.818
Model 2	0.609	0.567	0.555	23.990	<0.001	47.676	–0.579*	35.621*	0.768*	–	–	–	–
Extension													
Model 1	0.712	0.607	0.583	27.873	<0.001	97.362	–0.500*	42.964*	0.335*	0.075	–0.032	8.763	–2.063
Model 2	0.717	0.614	0.607	26.706	<0.001	81.038	–0.470*	46.897*	0.292*	–	–	–	–
Abduction													
Model 1	0.723	0.522	0.500	27.214	<0.001	63.247	–0.471*	36.549*	0.662*	1.305	0.088	3.978	–2.560
Model 2	0.757	0.594	0.577	25.733	<0.001	46.103	–0.425	36.065*	0.670*	–	–	–	–
Adduction													
Model 1	0.711	0.474	0.444	33.271	<0.001	111.679	–0548*	34.178*	0.355*	2.107	–0.072	15.640	–4.359
Model 2	0.786	0.544	0534	32.338	<0.001	75.975	–0.501*	35.642*	0.267*	–	–	–	–
Internal rotation													
Model 1	0.637	0.505	0.477	33.233	<0.001	98.314	–0.446*	37.716*	0.513*	–0.420	–0.103	13.161	–2.795
Model 2	0.733	0.600	0.591	32.880	<0.001	83.992	–0.463*	41.944*	0.385*	–	–	–	–
External rotation													
Model 1	0.610	0.472	0.443	28.015	<0.001	95.826	–0.346*	28.134*	0.490*	–1.674	0.016	9.553	–3.437
Model 2	0.699	0.559	0.550	27.779	<0.001	77.902	–0.386*	30.991*	0.347*	–	–	–	–
Elbow–flexion													
Model 1	0.681	0.538	0.507	51.244	<0.001	209.758	–0.694*	48.042*	0.426*	–3.003	–0.132	29.527†	–9.023
Model 2	0.696	0.563	0.544	51.997	<0.001	144.028	–0.785*	53.613*	0.341*	–	–	–	–
Extension													
Model 1	0.641	0.406	0.394	38.535	<0.001	108.184	–0.458*	35.304*	0.517*	–3.144	–0.047	19.776	–3.705
Model 2	0.659	0.512	0.502	36.818	<0.001	78.878	–0.495*	35.696*	0.443*	–	–	–	–
Wrist–flexion													
Model 1	0.646	0.398	0.365	29.026	<0.001	81.096	–0.354*	22.799*	0.443*	–0.671†	–0.038	9.465	–2.837
Model 2	0.672	0.477	0.467	28.216	<0.001	65.408	–0.358*	24.400*	0.341*	–	–	–	–
Extension													
Model 1	0.594	0.444	0.409	32.768	<0.001	86.757	–0.322*	15.149*	0.555*	–4.355†	0.042	28.150†	–2.475
Model 2	0.624	0.480	0.468	32.601	<0.001	64.032	–0.371*	17.174*	0.418*	–	–	–	–

*P<0.05; †Variable with P>0.05 with the removal of regression; Model 1: Initial regression model containing variables with potential to determine the peak torque of isometric contraction in the literature; Model 2: Regression model keeping only variables with P<0.05 in model 1; r: Correlation coefficient; r^2: Coefficient of determination; $r^{2adjusted}$: Coefficient of determination adjusted for chance; SEE: Estimated standard error; ß: Standardized coefficient.
